# Recurrent pericarditis in a patient with Bardet–Biedl syndrome: A case report

**DOI:** 10.3389/fped.2022.976751

**Published:** 2022-11-30

**Authors:** Angela Mauro, Francesca Casini, Emanuele Chittano Congedo, Sara L’assainato, Francesca Pinto, Valentina Ansuini, Ruggiero Mascolo, Alice Pedroli, Luca Bernardo

**Affiliations:** ^1^ Department of Pediatrics, Rheumatology Unit, ASST Fatebenefratelli-Sacco, Milano, MI, Italy; ^2^ Department of Pediatrics, Vittore Buzzi Children Hospital, Milano, MI, Italy; ^3^ Department of Pediatrics, ASST Fatebenefratelli-Sacco, Milano, MI, Italy; ^4^ Department of Medicine, ASST Fatebenefratelli-Sacco, Milano, MI, Italy

**Keywords:** Bardet–Biedl syndrome, recurrent pericarditis, anakinra, autoinflammatory disease, children

## Abstract

Bardet–Biedl syndrome is a rare autosomal recessive disorder characterized by rod-cone dystrophy, renal dysfunction, obesity, learning difficulties, hypogonadism, polydactyl, and many other minor features that can affect the cardiovascular, locomotive, neurological, and endocrine systems. We report the case of a 16-year-old boy affected by Bardet–Biedl syndrome who presented with recurrent pericarditis with an optimal response to treatment with Anakinra. To our knowledge, this is the first description of an association between Bardet–Biedl syndrome and recurrent pericarditis.

## Introduction

Bardet–Biedl syndrome (BBS) is a rare autosomal recessive genetic disorder that belongs to the group of ciliopathies. According to the modified diagnostic criteria by Beales et al. (Table 1) (1), at least four primary features, or three primary features and two secondary features, are required to make a diagnosis. The syndrome can be suspected as early as during pregnancy, by the presence of polydactyly and structural renal abnormalities during prenatal imaging (2). The suspicion can be confirmed by genetic analysis. As for cardiovascular anomalies, aortic stenosis, situs inversus, left ventricular hypertrophy, patent ductus arteriosus, and unspecified cardiomyopathy may be found (3). Associations between BBS and recurrent pericarditis (RP) have not been described in the literature thus far. Herein, we discuss the case of a 16-year-old child affected by BBS with RP treated with anakinra.

**Table 1:** Diagnostic criteria for Bardet–Biedl syndrome (BBS).

Primary features	Secondary features
Retinal dystrophy	Nephrogenic diabetes insipidus
Polydactyly	Developmental delay
Renal structural abnormalities	Speech defects/delay
Hypogonadism in males	Mild spasticity (mainly lower limbs)
Obesity	Imbalance/poor coordination/ataxia
Learning difficulties	Diabetes mellitus
	High arched palate/missing teeth/dental crowding/short roots
	Brachydactyly/syndactyly
	Astigmatism/cataracts/strabismus
	Hepatic fibrosis
	Left ventricular hypertrophy/congenital heart disease.

## Case presentation

A. is a Tunisian 16-year-old boy with BBS characterized by retinitis pigmentosa, polydactyly of the left foot, kidney cysts, and learning difficulties. His parents were not consanguineous, and, in his family, there are no other cases of BBS. He presented with the first episode of pericarditis at the age of 12 years, which was successfully treated with only colchicine (1 mg/day) at another hospital in Tunisia. An echocardiography was performed that showed a pericardial effusion of 4–5 mm. During this episode, a pericardiocentesis was not carried out. He continued colchine for 3 months. Later, in Italy, he presented with three further recurrences of pericarditis, at intervals of 1 year, treated with colchine (0.5 mg/day) and ibuprofen (600 mg/day) three times a day, which was progressively decreased to 200 mg/day twice a day. During the last episode, due to the presence of a large pericardial effusion, he underwent a pericardiocentesis with the evacuation of 900 ml of serum-hematic liquid. The culture and cytological examinations of the pericardial fluid were negative. He was treated with colchicine (1 mg/day) and ibuprofen (1,800 mg/day), which were progressively reduced to 600 mg/day. The patient came to our attention at the age of 16 years for retrosternal pain. On his first visit, his blood pressure was 130/90 mmHg with negative cardio-thoracic and abdominal physical examinations. Blood tests were carried out [complete blood count, erythrocyte sedimentation rate (ESR), PCR, troponin, liver and kidney function, electrolytes], which were in the normal ranges. An echocardiogram showed the presence of a minimal effusion of the free pericardium wall (5 mm) with the presence of fibrin. He was treated with colchicine (1 mg/day), indomethacin (150 mg/day, gradually tapered to 75 mg/day), and prednisone (5 mg/day). After 2 months, he returned to our department with chest pain, which worsened with movement. On physical examination, the patient was in fairly good general condition, his blood pressure was 150/90 mmHg, and his heart rate was 110 beats/minute. The cardio-thoracic and abdominal physical examination results were negative. Blood tests were carried out and showed an increase in inflammation index [C-reactive protein (CRP) 170 mg/L, ESR 80 mm/h) and white blood cell count 10,750/mm^3^, and neutrophils 7,920/mm^3^. Anti-nucleus antibodies, ENA antibodies, anti-phospholipid antibodies, fecal calprotectin, viral serologies, and Mantoux tests were all negative. Both chest x-ray and electrocardiogram were negative, and an echocardiogram revealed the presence of pericardial effusion (20 mm). Since the pericarditis was cortico-dependent and colchicine resistant, he started therapy with Anakinra (100 mg/day). The patient quickly showed an improvement in clinical, laboratory, and instrumental conditions. For this reason, prednisone, indomethacin, and colchicine were progressively tapered and finally discontinued. The patient had no other relapses. At his last visit, the patient appeared in good general condition; he had no dyspnea or thoracic pain. He underwent blood tests, electrocardiogram, and echocardiography, all with results in range. In fact, he continues to take Anakinra at a dose of 100 mg/day.

## Discussion

BBS is a rare autosomal recessive multisystem ciliopathy. The incidence of BBS is 1 in 150,000–160,000 in Europe and North America (2) but seems to be higher in Kuwait and the Faroe Islands, where it can reach 1:17,000 and 1:3,700, respectively (4).

BBS is caused by a dysfunction of the gene coding for proteins that are implicated in the function of the primary cilia and in important signaling pathways (5).

Genetic confirmation of the diagnosis can be obtained by sequencing BBS genes. However, access to such sequencing is usually restricted due to genetic heterogeneity and excessive cost, especially in low-income countries. In fact, 21 causing genes (BBS1–BBS21) have been identified and, in particular, BBS1 and BBS10 are the most frequently involved.

In the literature, cardiac involvement in BBS is usually linked to congenital heart disease but acquired heart diseases, such as pericarditis, are extremely rare. Moreover, to date a correlation between BBS and rheumatological or autoinflammatory diseases has not been described.

Our patient presented four primary features that were in accordance with the classification by Beales et al., and a history of RP.

According to the European Society of Cardiology, pericarditis can be defined as a clinical and acute inflammatory pericardial syndrome and involves at least two of the following criteria: pericardial rubs (30% in the pediatric population); chest pain (90%–95% in the pediatric population); ECG changes (40%–50% in the pediatric population); pericardial effusion (70%–80% in the pediatric population); pericardial inflammation at imaging, and elevated inflammatory markers.

Acute pericarditis can reappear and can lead to RP, described as a recurrence of acute pericarditis after a first episode with a symptom-free interval of at least 4–6 weeks (6). In children, about 70% of cases of RP are regarded as idiopathic and are usually a diagnosis of exclusion (7). Other possible causes of RP are infections (adenovirus, enterovirus, influenza A, cytomegalovirus, human herpesvirus-6, parvovirus, Epstein–Barr virus), connective tissue diseases, vasculitis, sarcoidosis, inflammatory bowel diseases, autoinflammatory diseases such as Familiar Mediterranean Fever, TNF receptor-associated periodic syndrome, or post-cardiac injury (7).

The exact pathogenesis of RP is still unclear and potentially multifactorial. The main hypothesis is that RP is caused by an interaction between genetic predisposition, environmental factors, and the immune system. Triggers of recurrence are still unknown. Some studies have assessed a loss of immune tolerance toward pericardial antigens in the initial episodes, which can cause other relapses, suggesting a central role in adaptive immunity and autoimmunity (8, 9).

Contextually, Tsyklauri et al. suggested that patients with BBS have a higher prevalence of autoimmune disorders as a result of transport complex BBSome dysfunction, which also regulates the development and homeostasis of B cells leading to an increased number of immature cells and few mature B cells. In addition, as the BBsome is involved in leptin signaling, which regulates the immune system and prevents overreacted immune responses, its deficiency leads to a dysfunction of immunity (10–12).

In our patient, viral serologies, autoantibodies, and Mantoux test were all negative. He underwent clinical (chest pain), inflammatory (elevated CRP), and echocardiographic (pericardial effusion) tests, which produced evidence of the presence of pericarditis. The episodes were all separated by at least 2 months, with a complete resolution of clinical and instrumental cardiac findings with anakinra, suggesting that unidentified autoinflammatory-mediated mechanisms can play a role in RP.

Tombetti et al. reported that phenotypes of RP with a remarkable response to anti-interleukin 1β (anti-IL1β) therapies, such as anakinra, could have the same pathogenesis as other autoinflammatory diseases. IL1β represents a fundamental marker of the immune pathways, such as the last step of the activation of inflammasome (7).

The excellent response to treatment with anakinra in our patient reinforces the hypothesis that many pediatric cases of RP could have an underlying unknown autoinflammatory mechanism.

Moreover, anti-IL1β could be an effective alternative treatment for those patients with RP with frequent relapses with other therapies, such as colchicine or steroids.

However, we did not perform genetic screening for anti-inflammatory diseases.

To our knowledge, this is the first case of BBS associated with RP. This relationship is particularly interesting as it indicates a new possible feature associated with BBS.

Therefore, physicians should be aware of a pericardial involvement in patients with BBS. Further studies are needed to confirm this relationship and the mechanisms behind this phenomenon, especially in children.

## Conclusion

We presented a case of RP in a child with BBS, who optimally responded to anakinra, suggesting a probable autoinflammatory origin of RP.

Is RP a new manifestation of BBS or a form of autoinflammatory pericarditis associated with it?

## Data Availability

The raw data supporting the conclusions of this article will be made available by the authors, without undue reservation.
